# Adapting Cure Violence for Southwest Philadelphia: A Pilot Implementation Evaluation of the Penn Community Violence Prevention Program

**DOI:** 10.1177/26334895261450993

**Published:** 2026-09-03

**Authors:** Sara Solomon, Ruth Abaya, Kathyrn C. Kaplan, Melissa Davey Rothwell

**Affiliations:** 1 14640 Department of Emergency Medicine, Penn Injury Science Center, University of Pennsylvania, Philadelphia, PA, USA; 2 14640 Health Alliance for Violence Intervention and Children's Hospital of Philadelphia, Philadelphia, PA, USA; 3 Mary Ann & J. Milburn Smith Child Health Outcomes, Research and Evaluation Center, 2429 Stanley Manne Children's Research Institute, Ann & Robert H. Lurie Children's Hospital of Chicago, Chicago, IL, USA; 4 25802 Health, Behavior, and Society, Johns Hopkins University, Baltimore, MD, USA

**Keywords:** adaptation, co-creation, interventions, community based, mixed methods (qualitative and quantitative) evaluation, evaluation, evidence informed

## Abstract

**Background:**

Community violence interventions (CVIs) aim to reduce firearm-related harm through strategies that center community engagement, credible messengers, and tailored outreach. Despite increasing adoption, few studies use implementation science methods to assess the implementation of adapted CVI models in new contexts. This pilot evaluation describes the implementation of the University of Pennsylvania Community Violence Prevention (PCVP) program, an adaptation of Cure Violence (CV) implementation strategies co-developed with community stakeholders. We assessed implementation outcomes and short-term participant-level impacts to inform future scale-up.

**Method:**

Using a type 3 hybrid implementation-focused design, we conducted a mixed-methods evaluation over an 18-week pilot period. Guided by the RE-AIM framework and Proctor's implementation outcomes, we examined reach, fidelity, acceptability, and appropriateness. Administrative data, staff-reported checklists, and geospatial outreach analyses were triangulated with survey data from 18 high-risk participants, informed by the COM-B model. Quantitative data were analyzed descriptively and using Pearson correlations; qualitative responses underwent rapid thematic analysis.

**Results:**

The PCVP team (two full-time, two part-time outreach workers, and one manager) engaged 38 participants, reaching 75% of target caseloads and covering 70% of shooting incidents within two blocks and two weeks. Fidelity was high for case management and community engagement, but low for violence detection/interruption due to staffing limitations. Participants reported high acceptability and appropriateness of the intervention (M = 4.39, SD = 0.98), with COM-B scores indicating strong engagement across capability, opportunity, and motivation domains (M = 25.64/30). Correlations revealed significant links between social/physical opportunity and motivation (r > .70, p < .01).

**Conclusions:**

This study demonstrates the feasibility and acceptability of a community-adapted CVI model. High acceptability and engagement support continued implementation, though limitations in fidelity to violence interruption underscore the need for targeted staffing and funding. Context-sensitive adaptation, robust community partnerships, and longer-term implementation research are essential for sustaining and scaling CVI efforts.

## Plain Language Summary

Study about adapting a community violence program to better support people at highest risk and keep neighborhoods safer

**Why was the study done?:**

Community gun violence harms health and well-being. Cities often use Community Violence Intervention programs, such as Cure Violence, which employ trusted community members to support people at highest risk for violence. Results vary by place, so it's important to see how the model works on the ground and which parts matter most. This study examined an adapted Cure Violence pilot to learn who was reached, how well core activities were delivered, and what short-term benefits participants reported.

**What did the researchers do?:**

An 18-week pilot ran in one neighborhood. Data came from program records, weekly staff checklists, and a participant survey. The team assessed:

- **Reach:** how many people were served, who they were, and where outreach occurred.
- **Implementation:** fidelity to case management/mentoring, community engagement, and conflict detection/mediation.
- **Effectiveness (short term):** participants’ capability, opportunity, and motivation to make positive changes.

**What did the researchers find?:**

- **Who was served:** 38 people supported by two full-time and two part-time outreach workers, supervised by a program manager; most met multiple highest-risk criteria.
- **Outreach alignment:** About 70% of shootings in the focus area were followed by outreach on nearby blocks within two weeks.
- **Supports provided:** Job help, mental health services, and practical needs such as obtaining a drivers license or birth certificate; participants had about 11–30 documented supports.
- **Delivery fidelity:** High for case management/mentoring and community engagement; low for conflict detection/mediation (active mediation in only 3 of 18 weeks), largely due to staffing.
- **Short-term benefits:** Among 17 survey respondents, average scores were high—especially for motivation and skills/ability. Participants cited emotional support, hope, and help with jobs and daily challenges.

**What do the findings mean?:**

The program effectively engaged individuals at high risk for violence and delivered strong mentoring and community engagement. Due to inadequate staffing and other unknown factors, we were unable to deliver all components of the intervention. However, the program was still able to deliver meaningful results for high-risk participants. We also developed evaluation tools for other programs to use. Further research should investigate whether implementing the full model, including violence detection and interuption, has the greatest impact and assess the ripple effects beyond individual participants to the broader community.

## Background and Introduction

Community gun violence—defined as fatal and nonfatal firearm-related injury occurring primarily in public spaces—is a significant public health concern with broad physical, social, and mental health impacts. It disproportionately affects African American/Black and Latino communities in urban areas with concentrated disadvantage (Bancalari et al., 2022). Often likened to a contagion, community gun violence spreads across neighborhoods and peer groups, with poverty, limited educational opportunities, and disinvestment acting as vectors (Slutkin, 2013; Slutkin, 2014).

Viewing community violence through contagion theory reinforces its preventability and the need to disrupt transmission. Two strategies commonly highlighted in the literature and adopted by many cities include engaging local leaders and collaborating with residents affected by violence (Buggs, 2022; Schleimer et al., 2024; Solomon et al., 2025). These approaches are known in scientific and practice communities as community violence interventions (CVIs), defined as “approaches that use evidence-informed strategies to reduce violence through tailored community-centered initiatives” (The CVI Ecosystem, n.d.).

CVIs position those most affected as experts and often employ *credible messengers*—individuals with lived experience (e.g., gang involvement, street violence, or incarceration)—to influence those at highest risk (Szkola & Blount-Hill, 2025). National organizations (e.g., HAVI, NICJR, CBPS Collective, Cities United) support the scaling of CVIs by providing resources and technical assistance to implementing communities (*Coalition to Advance Public Safety*, n.d.).

### Cure Violence

One of the most widely replicated and studied CVIs is Cure Violence (formerly Ceasefire), which is grounded in contagion theory and seeks to reduce violence by interrupting transmission among individuals at highest risk (Cure Violence Global: The Cure Violence Approach, n.d.). Its original evaluation documented reductions in violence in “most” of the intervention neighborhoods (Skogan et al., 2009), and an additional 19 communities globally have formally adopted Cure Violence as of February 2025 (*Cure Violence Global History*, n.d.). In a scoping review, Solomon et al. (2025) found that among 20 studies that measured effectiveness, defined as changes in fatal or nonfatal violence, 5 (25%) concluded Cure Violence was effective, 10 (50%) reported mixed results (decreases in some neighborhoods and no change or increases in others), and 5 (25%) concluded the program had no effect or increased violence. These mixed findings highlight why Cure Violence is a “promising practice” (Pugliese et al., 2022) and underscore the importance of examining how programs are implemented and adapted to local resources, needs, and preferences.

### Adaptation of Cure Violence: Setting the Stage for Measuring Implementation

**Figure 1.**
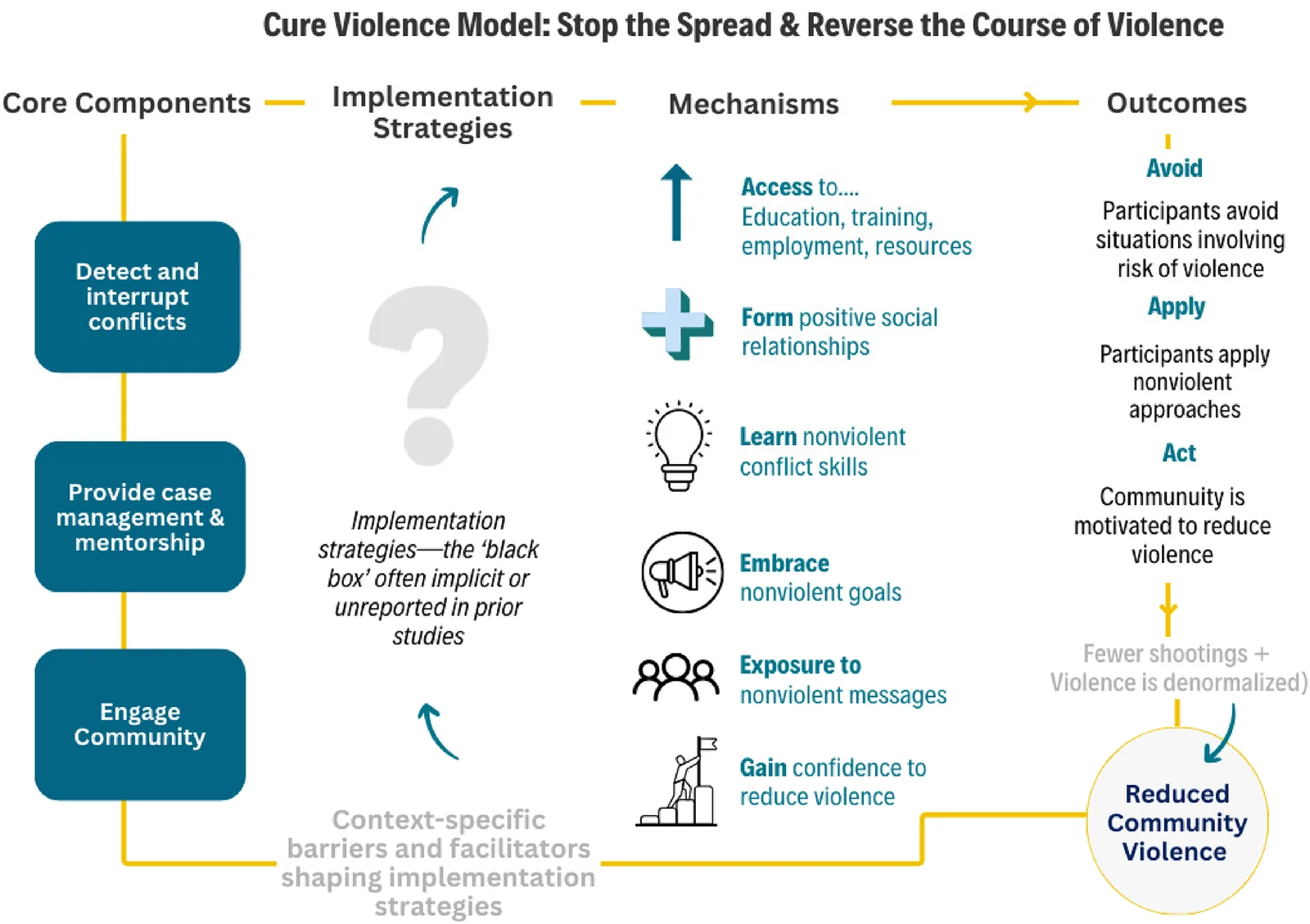
Core cure violence components and context-informed implementation strategies.

Although Cure Violence is often described as a standardized model, in practice it consists of three evidence-informed core components that are operationalized through implementation strategies that vary across sites (Figure 1). Prior replications frequently involve informal or undocumented changes to these strategies in response to staffing, safety, resource, and community context constraints, limiting opportunities for cross-site learning and cumulative implementation knowledge. In contrast, in this setting we used a structured, community-engaged adaptation process to specify and tailor the implementation strategies used to deliver the core components**,** making those strategy-level modifications explicit and systematic, awhile preserving the intended functions of the Cure Violence components.

Specifically, we employed a phased, systematic process to adapt the model, drawing from implementation mapping (Fernandex et al., 2019) over a 15-month period. This involved PCVP staff and a Community Advisory Board (CAB) working together to identify contextual barriers and facilitators of implementation and then tailoring implementation strategies to the local context. As part of this, we identified “priority” implementation strategies (Bunger et al., 2017; Proctor et al., 2013) for each core component, while maintaining fidelity to the core components of the intervention: interrupting and detecting patterns of violence; engaging the community to change social norms; and working individually with people identified as at highest risk for violence. We drew from the Expert Recommendations for Implementing Change (ERIC) (Powell et al., 2015) to name strategies using a standardized taxonomy and worked with staff to define strategies related to their position and responsibilities. Staff received training before beginning the 18-week pilot implementation phase. This resulted in a co-created implementation blueprint with twenty priority implementation strategies across the three core components. Full details of the adaptation process are documented elsewhere (Solomon et al., 2026).

### Objectives

This paper presents the pilot evaluation findings of the adapted pilot intervention. Our evaluation was guided by the RE-AIM framework (Kessler et al., 2012), with a focus on outcomes related to reach, effectiveness, and implementation (e.g., acceptability, appropriateness, fidelity). Our primary research questions guiding the evaluation were: (a) What is the reach, fidelity, acceptability, and appropriateness of the Cure Violence pilot in Southwest Philadelphia? (b) What is the short-term impact of the adapted program participants’ capabilities, opportunities, motivations, and behaviors? The findings provide actionable steps for tailoring the implementation of the local model and offer tools and recommendations for fostering participant involvement in violence prevention initiative evaluations across CVIs.

### Conceptual Framework/Approach

**Figure 2.**
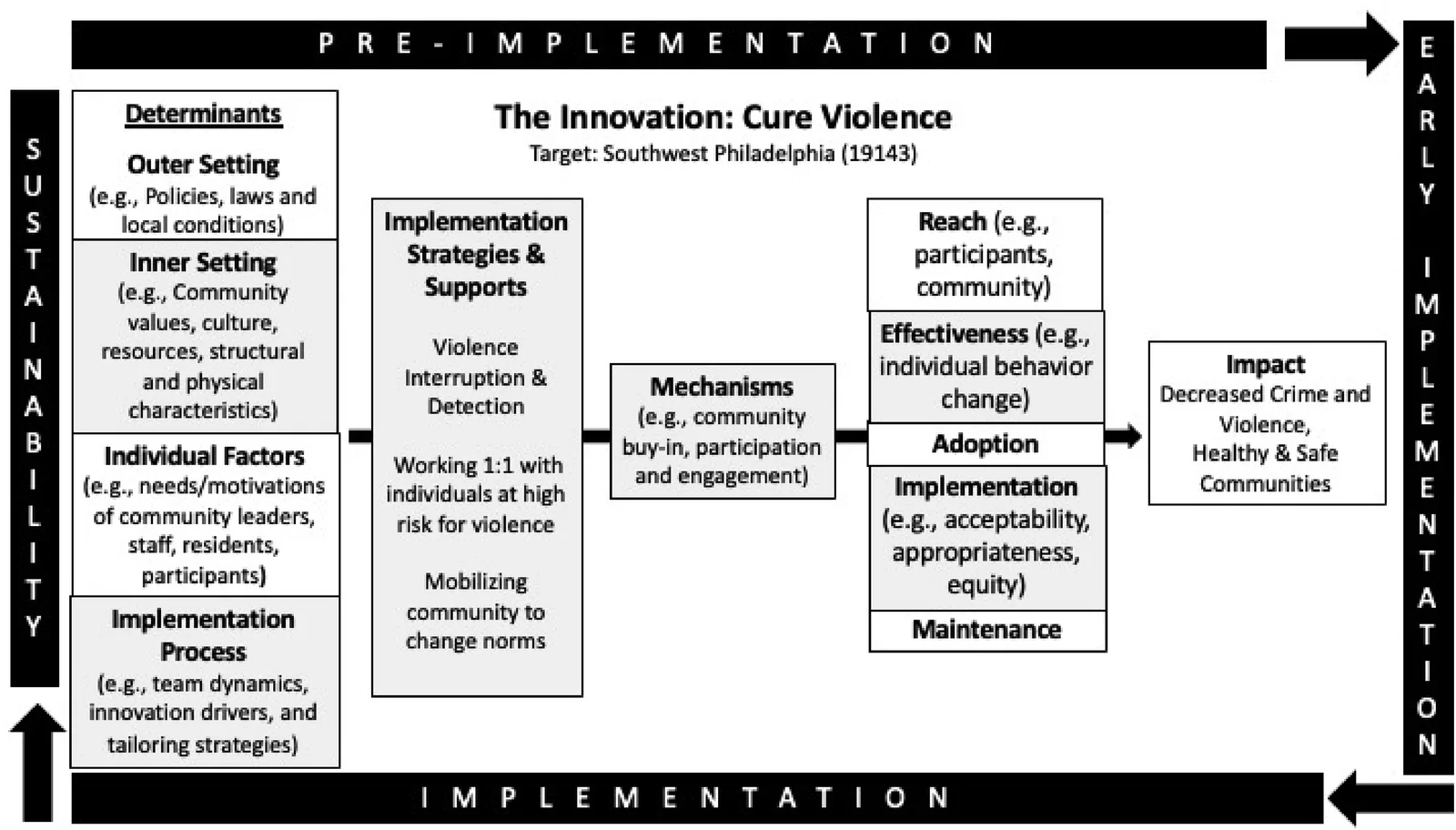
Conceptual framework.

Our approach is grounded in implementation science (Warren & Mauk, 2019, Moulin et al., 2019), principles of community-based participatory research (CBPR) (Coombe et al., 2020; Israel et al., 2005; Sánchez et al., 2021), and equity-centered design (ECD) (Christine Ortiz, n.d.)​. CBPR and ECD offer complementary principles for partnering with communities to co-develop interventions and evaluations that are responsive to local priorities, address structural inequities, and support sustainable, culturally responsive solutions. In practice, we operationalized these principles by valuing lived experience as expertise in our staffing model, centering community priorities and shared decision-making through a Community Advisory Board (CAB) and staff-led feedback cycles that shaped procedures, measures, and interpretation of findings to ensure local relevance and feasibility. As depicted in the conceptual framework (Figure 2), we hypothesized that using a community-engaged adaptation process tailored to the Southwest Philadelphia context would optimize program reach, implementation quality, and short-term participant outcomes, while also positioning the program for longer-term adoption and maintenance. Given the 18-week pilot timeframe, the current evaluation focuses on RE-AIM domains most appropriate for early implementation—reach, effectiveness (proximal outcomes), and implementation—while deferring adoption and maintenance for future study.

## Methods

### Study Design

To evaluate this pilot, we used a type 3 hybrid design (Curran et al., 2012) because the primary aim was to assess whether the adapted model could be delivered with fidelity, acceptability, and appropriateness in this setting, while secondarily examining short-term participant outcomes. We selected an implementation-first design (type 3) given documented cross-site variability in Cure Violence effects and the likelihood that outcomes are contingent on implementation strategies and context.

We collected qualitative (open-ended questions) and quantitative (surveys, administrative data) concurrently between October 2024 and February 2025. We then merged quantitative and qualitative responses to answer the research questions (Palinkas et al., 2011) We followed the Standards Reporting for Implementation Studies Checklist (StaRI) in writing the manuscript (Pinnock et al., 2017).

### Setting and Participants

The study took place within a community violence prevention program based at the University of Pennsylvania serving a high-poverty urban area with disproportionately high rates of crime and violence. The program operated within a broader CVI ecosystem in Philadelphia—including school-based supports, community organizations, social service agencies, and criminal justice–adjacent systems—that shaped service exposure and implementation conditions (Office of the Controller, n.d.).

Study participants included five staff and 18 consented participants, 17 of which completed surveys. All staff had lived experience, including living and working in the targeted geographic area, prior incarceration, prior violent injury, and former street and gang life. Consented program participants included those in weekly communication and receiving assistance from an assigned outreach worker during the pilot adaptation period. They also lived or attended school in the targeted geographic area or received services.

During the recruitment period, 38 individuals were actively receiving PCVP services and met the program's “high-risk” criteria (≥3 of the following: history of shooting, violent assault, or incarceration; gang or street involvement; carrying a firearm; or membership in a peer network involved in violence or firearm possession). Of these 38**,** 32 (84%) were invited to participate in the research study; the remaining six were not invited because they were unavailable during recruitment (e.g., incarcerated or non-responsive to outreach). Of the 32 invited**,** 18 (56%) consented and enrolled in the study and 17 (45%) completed surveys, reflecting the realities of recruiting a highly mobile, justice-involved population.

Some activities, such as those measuring reach, were standard operating procedures among staff and were required as part of the outreach workers’ weekly responsibilities, as described in detail below. Participation in all activities beyond work responsibilities was voluntary. All participants had informed consent, and the study was approved by the Institutional Review Board (protocol 857352). PCVP participants were offered a $50 gift card upon completing a survey as a token of appreciation for their time.

### Data Collection and Measures

We applied the RE-AIM framework to measure the reach, effectiveness, and implementation at the individual, community and organizational levels. Administrative data related to implementation reach and fidelity were collected from CharityTracker, a secure case-management platform used to document participant contacts, services, and referrals in real time as part of routine operations (CharityTracker Homepage, n.d.). Outreach workers entered data daily following program protocols, with supervisory review by the program manager. We also relied upon self-reported implementation checklists that outreach workers completed on a weekly basis with similar oversight and spot checking by the program manager.

### Reach

We operationalized reach as the number and characteristics of active participants, the individual-to-outreach worker ratio, the amount and types of resources accessed (e.g., employment, housing, legal assistance), and the program's geographic reach. We classified a participant active if they maintained weekly contact with an outreach worker and had at least one documented assistance record. The expected caseload—3 to 5 individuals for part-time and 10 to 15 for full-time workers—served as the denominator for assessing caseload coverage. Publicly available police data were used to estimate the percentage of participants relative to violent assaults and firearm robberies in the 19143 zipcode during the pilot period (Philadelphia Police, n.d.).

To assess uptake of services, we used self-reported participant data from a survey administered at the end of the 18-week pilot implementation period. Participants reported the frequency with which they accessed items from a predefined list of services, with an open-ended “other” option. The list captured a range of supports aligned with participant-identified goals, including referrals for mental health or substance use treatment, housing relocation, and employment support. Outreach workers did not provide these clinical or social services directly; rather, they offered social support and facilitated referrals to help participants achieve their goals.

To assess geographic reach, we analyzed the alignment between outreach locations and shooting incidents. To do this, we used Python (Van Rossum & Drake, 2009) to preprocess outreach and shooting data and employed the MapQuest API (*MapQuest: MapQuest Developer Documentation, 2023*) to geocode both datasets. A successful outreach was defined as the team covering a street within two blocks of a shooting within 2 weeks after the incident occurred, indicating a timely response to violence.

Reach also assessed the number of participants classified as “highest-risk” or those that met at least three out of the following five criteria: gang involvement, a leadership role in a drug or street organization, a history of violent crime, a reputation for carrying a firearm, or association with peers who carry weapons. Additionally, individuals were classified as high-risk if they had been a recent victim of a shooting or violent assault or had a serious fighting history within the past 6 months (Goldstick et al., 2024).

### Implementation

We measured implementation by examining constructs guided by Proctor's implementation framework (Proctor et al., 2011) focusing on implementation fidelity, acceptability and appropriateness of the intervention. We also measured partnership outcomes related to meaningful stakeholder engagement (PCORI. Foundational Expectations for Partnerships in Research, 2024) from the CAB which is the focus of a consecutive paper.

Fidelity examined the extent to which the implementation strategies were delivered as planned (Proctor et al., 2011) and was assessed via a computed adherence score, calculated as the proportion of outreach workers who reported completing the respective strategy in each week using dichotomous (yes/no) response categories and averaged across each core Cure Violence component per week. These weekly scores were then averaged across 18 weeks to generate an overall adherence summary score for each component. Because opportunities for violence interruption were episodic, we first identified weeks in which a conflict was detected; within those weeks, we assessed adherence to interruption strategies using the checklist items. Findings were integrated with open-ended responses to identify barriers and facilitators.

We operationalized the acceptability and appropriateness of the intervention from the client perspective by adapting questions using the Acceptability of Intervention Measure (AIM) and the Intervention Appropriateness Measure (IAM) (Weiner et al., 2017). We used a five-point Likert scale and averaged scores across each construct. Open-ended responses elicited additional perspectives on helpful resources and general areas for improvement.

### Effectiveness

We used the participant survey to assess effectiveness by examining participant-level intermediate outcomes in the CV logic model (Butts et al., 2015), related to achieving prosocial behaviors (McMahon et al., 2013; Pfattheicher et al., 2022). Survey questions were guided by the COM-B model (Fuster et al., 2023; West & Michie, 2020) to evaluate changes in participants’ capability, opportunity, and motivation to engage in positive behaviors to meet goals. We also adapted survey questions from a more established CV program in Chicago relevant to the research question (Papachristos, 2025). Participants self-reported progress using both quantitative, Likert scale, and qualitative, open-ended questions. The survey is made available in the appendix.

### Analysis and Interpretation

We employed a convergent parallel mixed-methods design (Hill et al., 2019; Saldana, 2014) by analyzing quantitative data (administrative records and closed-ended survey components) and qualitative data (open-ended survey questions) concurrently and then merging the two for a deeper interpretation and discussion of the results. For quantitative data, we used descriptive statistics (e.g., means and standard deviations) from Likert-scale responses and calculated means and standard deviations for each domain, providing an overall measure of participant perceptions. We used dichotomous (yes/no) responses to report on frequencies and proportions. Individual scores were averaged across their respective items to ensure consistency in weighting across domains, and a total score was calculated by summing the domain-level means.

We followed Fuster et al. (2023) to analyze the capabilities, opportunities, and motivations of participants to analyze data related to effectiveness. To avoid giving more weight to domains where more items were incorporated, the six individual COM-B domain scores were calculated as the average of responses received within the individual items related to the domain. We calculated a total COM-B score by summing up the resulting six individual scores, with a potential range of 6 to 30, where higher scores denoted greater capability, opportunity, and motivation towards prosocial behaviors. For descriptive, exploratory analysis, we transformed domain scores into a categorical variable to denote high scores, using the cut-off of 4 or higher. However, total and domain scores were analyzed as continuous variables given the resulting distribution of the exploratory categorical variables. We computed mean and standard deviations for each item in the COM-B scale, as well as the total COM-B score and domain scores. Correlations between the six domains were assessed using Pearson's correlation coefficient (*r*) to evaluate relationships between capability, opportunity, and motivation factors.

For qualitative data from the participant surveys, we conducted thematic analysis (Braun & Clarke, 2006) using an integrated approach that combined inductive and deductive reasoning (Proudfoot, 2023, Ramanadhan et al., 2021). Specifically, we followed Hamilton's guidelines for rapid qualitative analysis by creating summary sheet with pre-determined domains guided by the RE-AIM and CFIR frameworks (Damschroder et al., 2022; Kessler et al., 2012) as well as an “other” category. Thematic summaries were organized into a matrix to identify themes and representative quotes which were integrated with quantitative findings prior to sharing preliminary results with PCVP staff and the CAB. Based on PCVP staff and CAB feedback, the primary author presented a final interpretation of results via a “gallery walk” (Frank & Krueger, 2025) and then finalized based on additional feedback to ensure that the findings reflected actual outreach practices and were meaningful to the target community.

## Results

### Reach

During the 18-week pilot period, 38 participants received support from a team of two full-time and two part-time PCVP outreach workers, overseen by a program manager. Seventy-five percent of workers met their expected caseload (10–15 participants for full-time; 3–5 for part-time). Participants represented approximately 15% of all recorded assaults and firearm-related robberies within the targeted police district during the implementation period.

All participants (Table 1) identified as Black or African American; the majority were male (71.43%) and between ages 25 and 34 (55.26%). A majority (65.78%) were on probation, parole, or house arrest. Risk factors were common: 60.53% had a history of violence (perpetration and/or victimization), 44.74% were known for carrying firearms, and 36.84% had documented gang involvement. Over 60% met three or more high-risk criteria.

**Table 1.** Characteristics of PCVP Participants (*N* = 38) During Pilot Period.

Characteristic	No. (%)	
Age, years		
15–19	9	(23.68)
25–34	21	(55.26)
35–44	8	(21.05)
Sex^a^		
Male	20	(71.43)
Female	8	(28.57)
Race		
Black or African American (non-Hispanic)	38	(100)
Legal Status		
Parole	1	(2.63)
None	15	(39.47)
Probation or House Arrest	22	(57.89)
Risk Assessment		
Recent victim of a shooting or violent assault	4	(10.53)
Major player in gang or street involvement	5	(13.16)
Gang involved	14	(36.84)
Reputation of carrying a gun	17	(44.74)
Peer groups carry a gun	21	(55.26)
Previous violent history	23	(60.53)
Assistance provided by Outreach Worker		
0–10	10	(26.32)
11–20	12	(31.58)
21–30	12	(31.58)
31+	4	(10.53)
PCVP Staff providing assistance		
Full-time Outreach Worker 1	8	(21.05)
Full-time Outreach Worker 2	12	(31.58)
Part-time Outreach Worker 1	7	(18.42)
Part-time Outreach Worker 2	8	(21.05)
Program Manager	3	(7.89)

a Missing data.

Participants received individualized support including assistance with employment, education, legal matters, housing, and transportation. Most (63.16%) received 11 to 30 documented acts of support during the 18-week pilot. Resource uptake was highest for employment (70.59%) and mental health services (41.18%), followed by social activities (29.41%) and driver's license support (11.77%).

Finally, there was alignment across space and time between shooting incidents in the targeted geographic area and outreach strategies (e.g., shooting response, canvassing) within 2 weeks of a reported shooting. Out of all recorded shooting incidents in the geographic focus area, 69.8% were followed by outreach within the defined two-block/2-week threshold.

### Fidelity

Data from 17 client surveys, 59 weekly checklists, and 18 weeks of administrative data (38 participants) revealed varying fidelity across Cure Violence (CV) components (Table 2). Fidelity was high for two core components: individual behavior changes and community engagement, but low for violence detection and interruption, which occurred in only 3 of 18 weeks. Weekly checklists showed high adherence to building trust and active listening (95.0%), resource connection and communication (91.8%), and goal planning (84.8%). Challenges included participant availability, transportation barriers, and misconceptions about the program as financial assistance rather than a comprehensive intervention.

**Table 2.** Fidelity (Adherence Scores) of Prioritized Implementation Strategies.

Strategy	Definition	Adherence Score
*Provide case management and mentorship*		
Establish and build trust	Outreach workers build trust with client from introduction through case management process.	95.0%
Practice active listening*	Outreach worker listens actively to participants’ needs without judgement.	95.0%
Create individualized plans/tailor strategies*	Outreach worker tailors goal plans to meet to individual client needs.	84.8%
Connect to resources	Outreach worker/case manager identifies resources to help client meet needs and goals.	91.8%
Communicate regularly	Outreach worker initiates communication with client at least three times a week (1X in person)	91.8%
*Engage Community*		
Face-to-face communication	When engaging participants and community participants prioritizes face-to-face contact with community residents and organizations.	72.6%
Consistent community presence	Outreach team canvasses the community on a weekly basis to create partnerships, identify individuals at high-risk and build a community presence	86.4
Promote network weaving*	Outreach team and program build on existing high-quality working relationships and a community coalition to promote information. sharing, collaborative problem-solving, and a shared vision/goal related to implementing the innovation.	88.9%
Demonstrate credibility	Outreach team rely on connections, lived experience (former history of street life or violence) to get intel, referrals and resources.	96.7%
Distribute educational materials	Outreach team distributes flyers and brochures about PCVP during weekly community canvassing that describes the program and contains contact information.	94.4%
*Violence Detection and Interruption*		
Ensure safety	Program manager creates a safety protocol that outreach workers follow to ensure safety measures are in place for each conflict mediation.	100%
Immediately de-escalate	Outreach worker or community partner aims to immediately de-escalate active conflict between individuals or groups.	88.9%
Build trust ahead of time	Outreach worker or community partner forms trusting relationships with groups and individuals at high risk for conflict before active conflict.	83.3%
Listen actively to each of the conflicting parties	Outreach worker listens to the needs of both parties in conflict without judgement or criticism.	55.6%
Tailor strategies to meet all parties needs	Outreach worker and PCVP staff tailor mediation strategies based on the conflict at hand.	83.3%
*General Program Success+*		
Access new funding*	Access long-term funding to expand and sustain staff and programming.	n/a
Hire “credible” individuals	Leadership hires outreach staff that has lived experience (e.g., from community, former history of street life or violence) that relates to the mission of the program and target community.	n/a
Create an implementation blueprint*	Create a blueprint for implementation along with implementation checklists for clarity of goals and procedures.	n/a
Identify and prepare champions*	Identify local opinion leaders including leaders of street organizations, school principals, politicians, and faith-based leaders.	n/a
Use data*	Analyze data on an ongoing basis to identify and adapt target areas and track changes in violent activity over time.	n/a

ERIC = Adapted from Expert Recommendations for Implementation Change (Powell et al., 2015).

Note: CV = Cure Violence; +General program success adherence not assessed.

Fidelity was also high in efforts to shift community norms. Outreach workers maintained strong credibility (96.7%), distributed educational materials (94.4%), attended community events (88.9%), and had a consistent presence (86.4%). Collaboration extended to schools, CV programs, survivor advocacy groups, and state representatives. However, time management and administrative burdens were noted, with supervisors recommending structured training in data entry to improve tracking.

Fidelity was low (11.4%) in violence detection and interruption, with violence detection and interruption strategies occurring in only 3 of the 18 weeks. When implemented during the 3 weeks, fidelity was high for safety measures (100%), de-escalation (88.9%), trust-building (83.3%), and tailoring strategies (83.3%), though listening to both conflicting parties was lower (55.6%). Follow-ups through calls, texts, and school meetings helped mediate conflicts, but barriers included participant resistance, difficulty accessing both parties, and limited school collaboration.

### Acceptability and Appropriateness

Participants reported high acceptability and appropriateness (M = 4.39, SD = 0.98; 5 = point scale). Participants particularly valued the emotional and mental health support: “my outreach worker helped me stay focused and provided many resources.” Employment assistance and practical aid such as food and transportation were particularly valued. Many participants emphasized the importance of having someone to talk to who understands their struggles without judgment and the role of peer support in problem-solving and maintaining stability.

One participant described the value of the program for him as “bettering myself, behavior in school and fighting.” Others noted that access to employment opportunities and staying engaged in productive activities was critical to their well-being. Overall, participants expressed that the program distilled a sense of hope—as one participant stated, “I’ve become a more positive person and [the program] gave me hope.” Other participants emphasized the value with comments such as “Just having a peer to confide in who not only listens but is very helpful with problem-solving” and “I need people by my side.”

Participants identified key areas for improvement, including the need for broader outreach and promotion, expanded resources and staff support, and better communication and transportation options. Transportation was often a challenge getting to and from job interviews or program activities. In addition, some participants expressed the need to talk to their outreach worker at a greater frequency. As one participant wrote—“more regular meetings and stuff.” These insights suggest that while the program is well-received, enhancements in accessibility and logistical support could further improve its impact.

### Effectiveness

A cross-sectional analysis of 17 PCVP participants showed strong engagement across all COM-B domains. The average total COM-B score was 25.64 (SD = 2.11) out of a possible 30, with an average domain score of 4.45 (SD = 0.68) on a five-point scale. Most participants scored ≥4.0 across domains (Table 3), and six participants demonstrated full engagement. Others scored high in at least five of the six domains.

**Table 3.** COM-B Scale Items and Mean Scores From Client Survey Respondents (*n* = 17).

OM-B Domain Scale Item^1^		Item Score Mean	SD
Capability—Physical (Knowledge)		4.46	0.50
The communication between me and my outreach worker has been consistent and clear.		4.63	0.50
I am aware of my goals that I set with my outreach worker.		4.38	0.62
Capability—Psychological (Skill)		4.26	0.48
I have the skills to achieve my goals.		4.50	0.52
My goals are easy to achieve.		4.07	0.96
I am capable of handling challenges or setbacks in my life.		4.06	1.06
My outreach worker regularly communicates with me to update me on my progress and next steps.		4.56	0.51
Opportunity—Social (Social influences)		4.33	0.76
My outreach worker has developed a personalized plan tailored to my specific needs and goals.		4.13	0.96
My outreach worker has helped you solve conflicts and problems (e.g., with family, school, POs, etc.)		4.25	1.06
My outreach worker has successfully connected me to resources that support my needs.		4.38	0.62
Opportunity—Physical (Environmental constraints)		4.39	0.62
My outreach worker has introduced me to people or groups that help me stay focused on positive change.		4.06	0.93
The resources provided to me have been useful in helping me stay on track toward my goals.		4.44	0.63
I have the resources I need to continue to achieve my goals.		4.38	0.81
The plan created for me is realistic and achievable.		4.50	0.63
Motivation—Reflective (Self-efficacy, plans)		4.56	0.45
Since participating in the PCVP program, I feel more motivated to make positive changes in my life.		4.67	0.49
I am confident in my ability to set and achieve goals.		4.44	0.51
I am focused on making positive changes in my life.		4.56	0.51
I am motivated to continue making progress.		4.63	0.50
Motivation—Automatic (Emotions, reinforcements)		4.53	0.41
I trust my outreach worker.		4.75	0.45
I feel comfortable discussing my needs and concerns with my outreach worker.		4.60	0.51
I feel heard and understood by my outreach worker		4.44	1.03
My outreach worker has had a positive impact on my life.		4.31	0.60
I am hopeful that I will continue to achieve my goals.		4.63	0.50

The highest-rated domains were reflective motivation (M = 4.56) and physical capability (M = 4.46). High mean scores were also observed in physical opportunity (M = 4.39) and social opportunity (M = 4.33). Psychological capability was the lowest-rated domain (M = 4.26).

Correlation analysis indicated strong and statistically significant relationships among key COM-B domains. The strongest correlation was between automatic motivation and physical opportunity (r = 0.75, p = .0008). Other strong correlations were observed between physical and social opportunity (r = 0.72, p = .0016), and between physical capability and social opportunity (r = 0.70, p = .0027). Due to the small sample size, we did not assess statistical significance for domain differences.

## Discussion and Limitations

This evaluation provides an assessment of the reach, implementation, and effectiveness of an adapted Cure Violence pilot program. A team of two full-time and two part-time outreach workers, along with one program manager, successfully implemented two of the three core Cure Violence components with fidelity. Outreach workers engaged individuals at high risk for violence, guiding them toward prosocial pathways, and actively engaged with the community to shift social norms around violence.

Outreach workers demonstrated high fidelity in engaging participants at elevated risk for violence. Their efforts were characterized by consistent use of core strategies such as building trust, active listening, linking participants to resources, and maintaining regular communication. This level of adherence is not unexpected, given the extensive training outreach workers received and several supportive contextual factors, including supervision by a staff member trained in social work and collaboration with a dedicated case manager from a partner organization. Additionally, the credibility of outreach workers—grounded in lived experience and community trust—likely contributed to the development and maintenance of strong participant relationships. Adherence was somewhat lower for two strategies: the development of individualized goal plans and consistent in-person engagement. Outreach workers and supervisors identified several contributing factors, including gaps in administrative skills, challenges with participant availability, and transportation barriers. Addressing these issues will be critical in future implementation efforts to strengthen participant goal setting and promote more consistent face-to-face interactions.

Similarly, outreach workers maintained high fidelity in community engagement efforts aimed at shifting social norms around violence. While the current study design does not support causal inference between community engagement and program outcomes, we hypothesize that community engagement may play a mediating role in enhancing reach, implementation quality, adoption, and sustainability. Future research should investigate the impact of community engagement on shifting norms related to violence, as well as examine whether robust engagement in program adaptation, implementation, and evaluation contributes to improved implementation fidelity and program effectiveness.

However, PCVP did not consistently implement violence detection and interruption, which likely contributed to the limited findings in this domain. Although detection and interruption are core elements of the Cure Violence model, programs vary in how they operationalize this function: some employ designated violence interrupters (VIs), whereas others embed interruption responsibilities within outreach worker roles (Solomon et al., 2025). During the pilot, interruption responsibilities were nominally embedded within outreach roles, but limited staffing and safety constraints reduced the program's capacity to detect conflicts early and engage both parties consistently. In our case, outreach staff responded to shootings but were not consistently able to mediate active conflicts or gather intelligence from individuals directly involved in street activity. This inconsistency likely reflected staffing capacity and safety constraints and may also relate to the changing nature of violence; staff noted that many conflicts increasingly originate on social media, which can reduce the visibility of early warning signs and limit opportunities for proactive mediation—even though violence is still enacted in public spaces and sustained community presence remains necessary.

These findings raise questions about the level of adherence needed for sustained violence reduction and the extent to which core components can be delivered under resource constraints. Given limited funding, PCVP placed greater emphasis on case management and community engagement during the pilot. While we observed short-term improvements, it remains unclear whether longer-term impact requires full implementation of the Cure Violence model or whether selected components may be sufficient.

In the few instances where PCVP conducted conflict detection and mediation, these efforts were made possible through a partnership with a local school's Youth Violence Reduction Initiative (School District of Philadelphia, n.d.), which enabled outreach workers to identify and address conflicts within the school setting. This collaboration accounted for all mediations conducted by the PCVP team and illustrates how community partnerships can be critical for adapting and extending intervention reach.

Taken together, these observations underscore the importance of both context-sensitive adaptation and strong community partnerships in implementing Cure Violence-inspired models. They also highlight the need for further implementation research to determine which components of Cure Violence are most critical under varying resource and contextual conditions, and how partial implementation may affect outcomes.

Findings also support the program's potential role in fostering prosocial behavior. High engagement scores suggest that participants generally felt motivated and equipped to make positive changes. The strongest-rated domains—reflective motivation and physical capability—may indicate a readiness to change and a sense of self-determination among participants. High ratings in opportunity domains further suggest the importance of resource availability and supportive environments in promoting behavior change.

The lower score in psychological capability could reflect challenges in self-efficacy or in applying new strategies consistently, highlighting a possible area for strengthening intervention support through targeted goal setting and confidence-building.

Correlations between opportunity and motivation domains emphasize the interconnectedness of these constructs. For example, participants who reported stronger emotional support also reported greater access to tangible resources, underscoring the importance of trusted relationships in improving engagement. These relationships may serve as both pathways to and reinforcers of prosocial behavior change.

While the short-term effectiveness findings from this pilot were promising it remains to be seen whether this adapted model will lead to sustained reductions in violence. A longer-term evaluation will be essential to assess the lasting effectiveness of this approach and to determine whether this adaptation reflects what is most needed and impactful in the Southwest Philadelphia context.

### Limitations

Several limitations should be considered when interpreting these findings. First, establishing a denominator for reach was challenging because PCVP engaged a dynamic, high-risk population; we therefore used a proxy denominator based on violent assaults and firearm-related robberies in the targeted area to contextualize reach.

Second, implementation fidelity relied on staff self-reported checklists, which may overestimate adherence. Although structured observation or third-party fidelity monitoring could strengthen implementation assessment, such approaches were not feasible given the dynamic, unpredictable nature of street outreach and related safety and logistical constraints.

Third, this non-randomized pilot lacked a comparison group and included a modest number of consented participants, limiting causal inference and generalizability. Effectiveness outcomes were restricted to short-term, individual-level self-reported measures, which may not reflect sustained behavior change or longer-term reductions in violence. Future studies should incorporate longer follow-up periods, larger samples, and experimental or quasi-experimental designs to strengthen attribution and assess durability.

Selection bias is also possible, as individuals who consented to research participation may have had greater short-term stability in communication or availability than those unreachable during recruitment. Transportation barriers reflected limited access to reliable transit and competing logistical or safety demands, while communication challenges were largely driven by frequent changes in phone numbers and intermittent service. Despite mitigation efforts—including transportation support, nearby community-based interview locations, and phone or videoconference options—some eligible participants could not be reached during the recruitment window.

A related limitation is potential spillover from the broader CVI ecosystem. Participants may have concurrently received supports from other community-, school-, healthcare-, or justice-adjacent programs, complicating attribution in this pilot evaluation; future studies should more explicitly characterize concurrent exposures and account for spillover in real-world CVI settings. Importantly, the broader CVI ecosystem should also be considered when interpreting these results and will be critical to account for in future implementation and scale-up efforts.

Finally, the evaluation would have benefited from integrating qualitative interviews conducted during Step 2 of the adaptation process. Although these data informed program design, their inclusion in the evaluation could have provided deeper insight into implementation mechanisms and contextual influences; future evaluations should incorporate these perspectives to strengthen interpretation.

## Future Directions

Building on the findings of this evaluation, we will apply key lessons learned to refine and strengthen the implementation of the program. We will revisit and revise workplans, incorporating additional staff training informed by adherence results. Special attention will be given to refining workplans and implementation checklists with a targeted focus on enhancing the violence detection and interruption component of the Cure Violence model. We will also explore additional implementation research to determine how the absence of fidelity around violence interruption influences broader outcomes. Furthermore, we will continue to invest in ongoing training and capacity-building for outreach workers to address identified barriers, including gaps in administrative skills and challenges with data tracking. These efforts will also emphasize strategies that received lower adherence ratings—such as face-to-face engagement and the development of individualized goal plans for individuals at highest risk of violence—to promote more consistent and effective implementation.

Future implementation research will be critical in optimizing program effectiveness. We plan to explore additional methods for assessing implementation fidelity, including structured observations and third-party evaluations, to complement the self-reported implementation checklists used in this study. Furthermore, given the small sample size and absence of a comparison group, future research should employ larger-scale evaluations utilizing experimental or quasi-experimental designs to assess long-term behavioral and community-level outcomes.

Building on the findings of this evaluation, our future research will seek to answer key questions about the most influential components of Cure Violence in driving implementation success and the underlying mechanisms that contribute to both effective and ineffective implementation. We will focus on participant engagement as a mechanism for optimizing reach, adoption, implementation, effectiveness, and sustainability of CV. Understanding how participant engagement influences program success will be essential in refining best practices for future CV adaptations.

## Client Survey

Welcome to the Penn Community Violence Prevention (PCVP) Participant Survey. This survey is designed to gather your feedback on the Penn Community Violence Prevention (PCVP) program. Your input will help us understand your client experience, how it has been implemented, and if it has been effective in helping participants meet their goals. Participation in this survey is voluntary. This means you can choose whether you want to be in the study. If you decide to participate or not to participate, there will be no impact on your role with the project. Because you are being asked to discuss your experiences you may disclose information that could be sensitive. We will not use your real name when we collect the data and <b>will not share your results with your outreach worker. Information that you provide will be used by project staff to communicate the results of the program. We will not link your name to any of the answers and only report findings from the aggregate. We use procedures to protect the confidentiality of anything you say.

After reviewing the confidentiality measures taken for this survey, do you agree to participate?

Yes, I agree to participate

No, I do not agree to participate

1. Hold old are you
2. How would you describe your gender?
  Boy/Man
  Girl/Woman
  Another gender identity :
3. Are you of Hispanic origin?
  No, not of Hispanic, Latino, or Spanish origin
  Yes, Mexican, Mexican American, or Chicano
  Yes, Puerto Rican
  Yes, Cuban
  Yes, another Hispanic, Latino, or Spanish origin
  Don't know
  Prefer not to say
4. Who is your primary outreach worker?
  Name 1
  Name 2….
  Don't know
  Prefer not to say
5. Which of the following best describes your race? (Check all that apply)
  Asian
  African American or Black
  Caucasian/White/European American
  Native American/American Indian/Alaskan Native
  Pacific 0Islander/Samoan/Hawaiian
  Multiethnic or mixed
  Don't know
  Prefer not to say
6. How many times have you met your outreach worker in person?
  0= I’ve never met my outreach worker
  1-2 times
  3-5 times
  6-10 times
  11-20 times
  More than 20 times
  Don't know
7. How many times have you had meaningful phone or text conversations with your outreach worker? (Meaningful conversations would not include short conversations for planning a meeting.)
  0= I’ve never had a phone or text conversation with my outreach worker
  1-2 times
  3-5 times
  6-10 times
  11-20 times
  More than 20 times
  Don't know

For the next set of questions, how much do you agree with the statement based on a scale of 1 to 5, with 1 strongly disagreeing and 5 strongly agreeing. If you do not want to answer leave blank.

8. My outreach worker regularly communicates with me to update me on my progress and next steps.
9. The communication between me and my outreach worker has been consistent and clear.
10. I trust my outreach worker.
11. I feel comfortable discussing my needs and concerns with my outreach worker.
12. I’ve tried to avoid my outreach worker.
13. My outreach worker listens to my concerns and respects my opinions.
14. I feel heard and understood by my outreach worker
15. I’ve lied or hid information from your outreach worker.
16. My outreach worker has developed a personalized plan tailored to my specific needs and goals.
17. The plan created for me is realistic and achievable.
18. My outreach worker has successfully connected me to resources that support my needs.
19. The resources provided to me have been useful in helping me stay on track toward my goals.
20. Your outreach worker has helped you solve conflicts and problems (e.g., with family, school, POs, etc.)
21. Which of the following resources did you from your outreach worker? Mental health services (e.g. counseling, therapy)
  Substance abuse service
  Education services (e.g. GED, high school support)
  Employment services (e.g. job placement, training)
  Obtaining a license (e.g. drivers, CDL)
  Fun activities (e.g. sports, music)
  Housing
  Other, (list): ________________________________
22. Which of the following resources did you participate in? select all that apply
  Mental health services (e.g. counseling, therapy)
  Substance abuse service
  Education services (e.g. GED, high school support)
  Employment services (e.g. job placement, training)
  Obtaining a license (e.g. drivers, CDL)
  Fun activities (e.g. sports, music)
  Housing
  Other, (list): ________________________________
23. How helpful the resources on a scale from 1 (not at all) to 5 (extremely helpful)?
  Not helpful at all
  slightly helpful
  Helpful
  Very helpful
24. What has been the most helpful part of the Penn Community Violence Prevention program for you?
25. Are there any aspects of the program that you think could be improved?
  __________________________________________

For the next set of questions, how much do you agree with the statement based on a scale of 1 to 5, with 1 strongly disagreeing and 5 strongly agreeing. If you do not want to answer leave blank.

26. My outreach worker has introduced me to people or groups that help me stay focused on positive change.
27. My outreach worker has had a positive impact on my life.
28. Since participating in the PCVP program, I feel more motivated to make positive changes in my life.
29. I am aware of my goals that I set with my outreach worker.
30. I am confident in my ability to set and achieve goals.
31. I have the skills to achieve my goals.’
32. I am capable of handling challenges or setbacks in my life.
33. My goals are easy to achieve.
34. I am focused on making positive changes in my life.
35. I am motivated to continue making progress.
36. I am hopeful that I will continue to achieve my goals.
37. I have the resources I need to continue to achieve my goals.
38. Which people or groups, if any, has your outreach worker introduced you to that help you stay focused on positive change?
39. To help me achieve my goals, I need the following resources:
40. I have the support I need to continue to achieve my goals: (select all)
  From my family
  From my friends
  From my community
  1. I need the following supports:
    __________________________________________

Thank you for completing this survey. Your responses will help us improve the PCVP program and ensure that we continue to meet the needs of clients like you.If you have any additional comments, please share them below:
